# Phylogeography and molecular species delimitation reveal cryptic diversity in *Potamolithus* (Caenogastropoda: Tateidae) of the southwest basin of the Andes

**DOI:** 10.1038/s41598-021-94900-3

**Published:** 2021-08-03

**Authors:** Gonzalo A. Collado, Cristian Torres-Díaz, Moisés A. Valladares

**Affiliations:** 1 Departamento de Ciencias Básicas, Facultad de Ciencias, Universidad del Bío-Bío, Avenida Andrés Bello 720, Chillán, Chile; 2 Grupo de Investigación en Biodiversidad y Cambio Global (GBCG), Universidad del Bío-Bío, Chillán, Chile

**Keywords:** Evolution, Phylogenetics

## Abstract

The species of the genus *Potamolithus* inhabiting the southwestern basin of the Andes are difficult to distinguish due to small size and similar shell morphology. Only *Potamolithus australis* and *Potamolithus santiagensis* have been traditionally recognized in this region, but the occurrence of several morphologically similar undescribed populations could increase the regional richness. Here we delimit described and potentially undescribed cryptic species of the genus using partial sequences of the mitochondrial cytochrome c oxidase subunit I (COI) gene. Network analysis and diversity indices inferred six highly differentiated haplogroups, many of them sympatric and widespread in the study area. Phylogeographic analyses suggest a scenario of recent diversification and the occurrence of multiple refuges during the successive Pleistocene glaciations. Phylogenetic analysis also recovered six major clades that showed no relationship with physiography. Species delimitation analyses consistently recognized three or four candidate species apart from *P. australis* and *P. santiagensis*. Divergence times indicate that speciation of Chilean *Potamolithus* began at the end of the Pliocene, probably driven by climatic rather than geographic events. Considering the high inter- and intra-basin genetic diversity, conservation efforts should be focused on protecting sympatric taxa in the basins with the highest species richness.

## Introduction

Species identification is increasingly important in biodiversity studies, conservation biology and natural heritage of the countries^1–4^. The high rate of species extinction as a consequence of habitat loss, pollution and global change, together with the enormous unknown biodiversity in some groups has led to the development of more effective and precise methods of taxonomic discrimination, which has been facilitated by technological advances^5^. It is now known that a portion of natural diversity is morphologically cryptic, so its identification depends on integrating molecular tools and traditional morphological approaches^6^.

A significant number of studies has shown that the evolutionary history of the biota in southern South America has been influenced by different geological processes and climatic events such as the Andean orogeny, marine introgressions, drainage reversals and glaciations^7–20^. The Andean uplift occurred from the Early Miocene generated the drainage divide between Chile and Argentina significantly impacting the distribution of genetic diversity of species, mainly the aquatic fauna^10,12,19,21^. The Patagonia Region suffered successive glaciations, with more than 10 major cooling events during the Pliocene and Pleistocene^22–25^. This intermittent melting and cooling of glaciers also had an impact on the underlying biota, leaving a genetic imprint on the phylogeography of several species^7,12,19^. Some of the strongest glacial events were the Great Patagonian Glaciation (GPG) c. 1 million years ago (Ma), several ancient glaciations collectively called the Pre-GPG occurred in the Lower/Middle Pleistocene (2.1–1.0 Ma) and three other cooling events called the Post-GPG that occurred in the Middle Pleistocene^25–28^. The boundary between the Piacenzian and Gelasian glaciations marks the boundary between the Pliocene and the Pleistocene, 2.59 Ma^29^. From the end of the Miocene the land south of 37°S was intermittently covered by ice sheets extending to Antarctica in the successive glaciations and whose greatest extension was reached during the Last Glacial Maximum (LGM) in the late Pleistocene, c. 0.025 Ma^24,25,30,31^. The tectonics and environmental processes generated expansion and retreat of forests, isolation of species into refugia, habitat fragmentation, extinctions, and the establishment of distributional limits promoted by transverse glacial tongues from the Andes that left open areas of secondary contact after melting^10,12,15,19,22^.

The superfamily Truncatelloidea Gray, 1840 is one of the most diverse groups of Caenogastropoda, with hundreds of genera^32–34^. Within this superfamily, the family Tateidae Thiele, 1925 is composed of minute freshwater snails that have a wide geographic distribution throughout the South Pacific and southern South America^35–38^. Tateid snails were traditionally included in the family Amnicolidae Tryon, 1863^35^, Hydrobiidae Troschel, 1857^39^ or Lithoglyphidae Troschel, 1857^37,40^, revealing inconsistencies in the classification; the monophyly of the Tateidae was recovered by Wilke *et al*^41^. Two native genera of this family have been recognized in South America, *Potamolithus* Pilsbry, 1896, which contains about 47 species^42^, and the monospecific genus *Strobellitatea* Cazzaniga, 2017^43^. A third genus is *Potamopyrgus* Stimpson, 1865, represented in Chile by the invasive species *Potamopyrgus antipodarum* (Gray, 1843)^44^. *Potamolithus* is a diverse genus of thick-shelled operculate snails comprising species that reach between 2.0 and 7.0 mm in length^35,45–49^. Although there are drawings or illustrations of the soft parts in some taxa, species taxonomy has been based mainly on characters of the external shell morphology^39,45,46,48,50^. In Chile only two species of the genus have been described based solely on external shell characters and operculum, *Potamolithus australis* Biese, 1944 and *Potamolithus santiagensis* (Biese, 1944). Apart from the original description, *P. australis*, typical of Llanquihue Lake in Chilean Patagonia, appears mentioned in a few later works and lists of species^47,51–54^ while López Armengol^55^ considered it as *nomen dubium*. *Potamolithus santiagensis*, originally described under the genus *Littoridina* Souleyet, 1852 from Dehesa Stream^36^ in central Chile and to which a population of El Yeso Spring was later added^56^, was subsequently included in the genus *Heleobia* Stimpson, 1865 but recently transferred to *Potamolithus* by Collado et al*.*^57^. These authors also added two populations from central Chile (Lo Carreño and El Colorado) to the range of *P. santiagensis*.

Mitochondrial DNA sequences generally constitute a powerful tool for species delimitation, particularly in groups that are difficult to resolve using morphological data^58^. The advent of species delimitation methods based on DNA sequences has provided a useful method to validate previously described species and identify new evolutionary units in a wide variety of taxa^59–64^. In the present study, based on the current taxonomic knowledge of the genus *Potamolithus*, we assessed the hypothesis that only two species exist along its distribution range in Chile. We performed phylogenetic and network analyses in a phylogeographic framework to study the biodiversity of the genus in the southwest basin of the Andes, both within and among species, including potentially cryptic species. We also used molecular species delimitation approaches to define species partitions considering already described species and new lineages in the group.

## Methods

### Specimen collection

**Figure 1:**
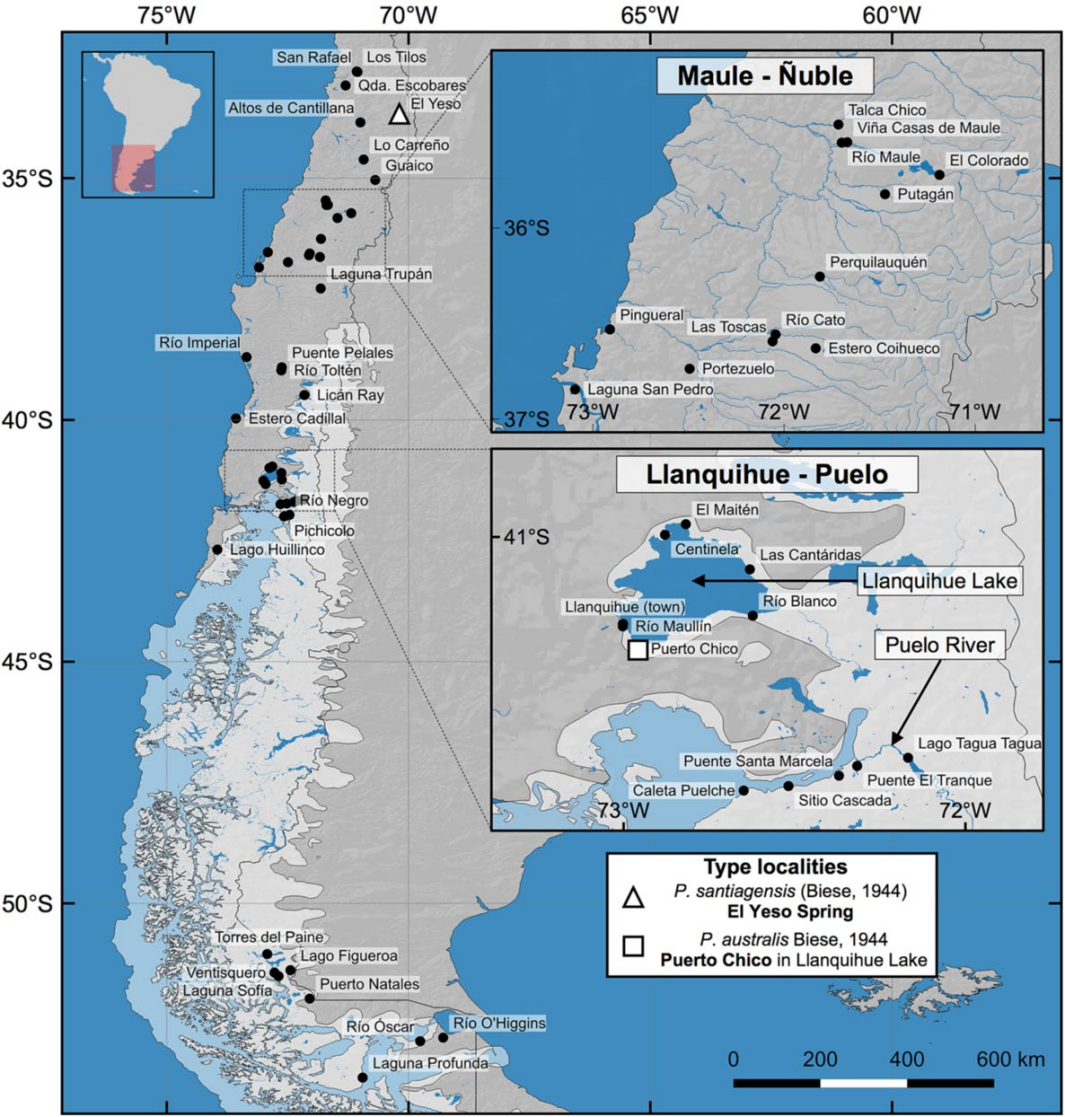
Sampling localities of *Potamolithus* in central and southern Chile of the present and other studies^44,57,65^. The areas of Maule-Ñuble and Llanquihue Lake-Puelo River are enlarged. The locality of El Yeso, historically assigned to *Potamolithus santiagensis*, is indicated by a white triangle. The type locality of *Potamolithus australis*, Puerto Chico in Llanquihue Lake, is indicated by a white square. The limits of the ice sheet during the Last Glacial Maximum in Patagonia were generated following different authors^24,30,31^. The map was created using QGIS Geographic Information System v3.4.9 (http://www.qgis.org). (Map: M.A. Valladares).

Snails were collected in 2015–2017 from multiple freshwater ecosystems located in central and southern Chile (Fig. 1; Table S1). All animals were collected using a hand sieve and then stored in absolute ethanol. Snail sampling was authorized by the Subsecretaría de Pesca y Acuicultura, Ministerio de Economía, Fomento y Turismo, República de Chile (Resolution No. 3285). The procedure for handling animals was approved by the Bioethics Committee of the Universidad de Valparaíso (Resolution No. 009–2013), institution with which the first author was associated at the beginning of the project. All methods were carried out in accordance with relevant guidelines and regulations proposed. The study was carried out in compliance with the ARRIVE guidelines (http://www.nc3rs.org.uk/page.asp?id=1357).

### Molecular analysis

For DNA extraction, PCR amplifications and sequencing of partial sequences of the mitochondrial cytochrome c oxidase subunit I (COI) gene we followed the methods described by Collado^44^ and Collado et al*.*^57,65^. Forward and reverse strands were corrected for misreads and merged into one sequence file using Sequencher v5.4.6 (Gene Codes Corporation, Ann Arbor, MI, USA). Sequences were aligned with MAFFT v7.470^66^ using the MAFFT online service for multiple sequence alignment^67^. To estimate genetic diversity of *Potamolithus* across the landscape we calculated the number of polymorphic sites (*S*), number of haplotypes (*H*), haplotype diversity (*Hd*) and nucleotide diversity (π) in DnaSP v5.10.1^68^. To visualize the relationships between haplotypes we constructed a median-joining network^69^ using the program PopART v1.7^70^. Genetic distances were estimated in MEGA X v10.2.6^71^.

Species delimitation in *Potamolithus* was studied using the Automatic Barcoding Gap Discovery (ABGD) method^72^ (a non-tree-based method)^59^, the multi-rate Poisson tree processes (mPTP) analyses^73^ and a Bayesian general mixed Yule coalescent (bGMYC) approach^74^. The ABGD method is based on genetic distances and automatically detects the gap between intra and interspecific divergence, which is then used recursively to delimit species hypotheses. The mPTP method delimits species trying to determine the transition from a between‐ to a within‐species process, but also incorporates different levels of intraspecific genetic diversity deriving from differences in either the evolutionary history or sampling of each species^73^. The GMYC method requires an ultrametric tree (or multiple trees sampled from a posterior distribution in the Bayesian implementation) and attempts to detect the transition between the branching pattern attributed to speciation and intra-species coalescent process. The ABGD method was performed on the online web server (https://bioinfo.mnhn.fr/abi/public/abgd/abgdweb.html) and was run with the default settings (relative gap width of 1.5; intraspecific divergence: between 0.001 and 0.1). For the mPTP and bGMYC analyses, phylogenetic reconstructions were performed previously using the Maximum Likelihood (ML) and Bayesian Inference (BI) algorithms, respectively. *Tatea huonensis* (Tenison-Woods, 1876) (GenBank accession number: JX970619^41^) was used as outgroup in both reconstructions. In addition, four species of *Potamolithus* from Argentina were included in the reconstructions: *Potamolithus agapetus* Pilsbry, 1911 (GB: KM220910^75^), *Potamolithus buschii* (Frauenfeld, 1865) (GB: KM220909^75^), *Potamolithus elenae* de Lucía & Gutiérrez Gregoric, 2016 (GB: KX397599, KX397600^76^) and *Potamolithus lapidum supersulcatus* Pilsbry, 1896 (GB: KX158843^76^). The ML reconstruction was performed in RAxML v8.2.12^77^ using the GTRGAMMAI model of nucleotide substitution and the node support was obtained by performing a bootstrap analysis of 1000 pseudo-replicates. The Bayesian reconstruction for the bGMYC method was performed in BEAST v2.6.3^78^ using the Relaxed Clock Log Normal model and a Coalescent Constant Population tree prior.

We applied two phylogeny‐aware approaches to delimit species in *Potamolithus*. First, mPTP analyses were performed using the online server (https://mcmc-mptp.h-its.org/mcmc/) and the ML tree from RAxML. For the mPTP method, after removing the outgroup, we performed two independent runs, each of 1,000,000 generations, sampling every 1000 generations and with a 10% burn-in. The second method was a GMYC model implemented in the package bGMYC v1.0.2^74^ in R v3.5.2 software^79^. For the analyses we used 100 random trees from the BEAST reconstruction (after removing outgroups). Simulations considered 50,000 generations, discarding 40,000 replicates, and setting a thinning every 100th generation. After preliminary analyses with varying parameters on a single tree, the upper and lower bounds of the Yule and coalescent rate change parameters were set to 1.0 and 0.5, respectively, and the upper prior threshold for the number of species was set at 100. Finally, all results were visualized using the package phytools v0.6–20^80^.

Divergence times were estimated in BEAST using a reduced data set of the haplotypes retrieved by DnaSP. For this analysis, a strict clock model was implemented using a substitution rate of 1.7% with a standard deviation of 0.34%^81^. For all BEAST analyses the site model was specified using bModelTest v1.2.1^82^, performing three independent analyses with 50 million generations each to infer a Maximum Clade Credibility Tree using a burn-in of 25%. A non-ultrametric Bayesian tree was reconstructed for the complete COI dataset using MrBayes v3.2.7^83^. The analysis used four parallel runs with 20 million generations each using a burn-in period of 25%. ML and BI reconstructions were performed in the CIPRES cluster of the San Diego Supercomputer Center^84^.

## Results

**Figure 2:**
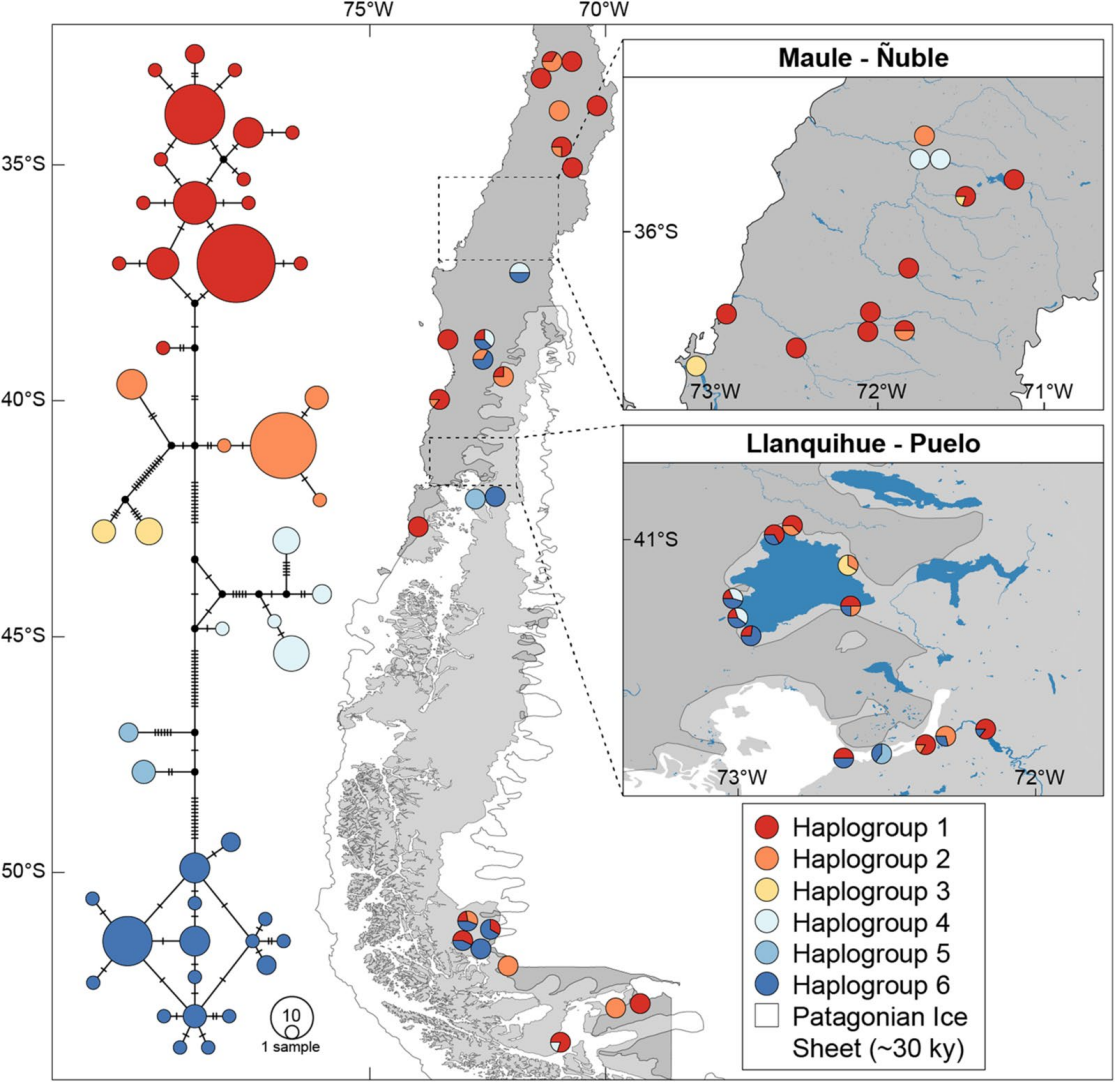
The median-joining haplotype networks of *Potamolithus* populations obtained in the present study. The areas of Maule-Ñuble and Llanquihue Lake and Puelo River are enlarged. The number of mutational steps among haplogroups is indicated by small bars. The limits of the ice sheet during the Last Glacial Maximum in Patagonia were generated following different authors^24,30,31^. The map was created using QGIS Geographic Information System v3.4.9 (http://www.qgis.org). (Map: M.A. Valladares).

The amplification of the COI gene in the Chilean *Potamolithus* (176 individuals) produced a fragment of 510 nucleotides in length. The mtDNA COI sequence alignment recovered a total of 86 polymorphic and 69 parsimony-informative sites, defining 47 haplotypes from 196 individuals considering our original sequences and those obtained from GenBank. The nucleotide composition in the complete dataset (excluding outgroup) was 38.7% A, 19.1% C, 16.1% G and 26.1% T. The best-fitting model of nucleotide substitution, as determined by bModelTest, was the HKY + I + G. The sequences showed a pattern of high haplotype diversity (*Hd* = 0.928) and nucleotide diversity (π = 0.0382). The diversity indices showed the co-occurrence of several genetic entities in the same locality (considering sites with *N* > 2). This was reflected by a large number of mutational steps and high nucleotide diversity within localities (Table S1). The median-joining network recovered six well-differentiated haplogroups without any evident latitudinal segregation (Fig. 2). Haplogroup 1 was composed of 16 haplotypes (89 individuals) detected in 33 out of the 48 localities analyzed, representing the largest latitudinal distribution ranging from Central Chile to the southernmost locality sampled in Patagonia (32° to 53°S). Haplogroup 2 was composed of five haplotypes (33 individuals) distributed in 17 localities from central Chile to Tierra del Fuego (32° to 52°S). Haplogroup 3 contained two haplotypes (7 individuals) presents in three localities in the southcentral area of Chile (35° to 41°S). Haplogroup 4 recovered five haplotypes (15 individuals) presents in seven localities in the south-central area (35° to 53°S). Haplogroup 5 was composed of two haplotypes (5 individuals) presents in two neighboring localities from the south of Chile (41°S), representing the narrowest distribution among all haplogroups. Haplogroup 6 was composed of 17 haplotypes (42 individuals) presents in 16 localities in the southcentral area of Chile (37° to 51°S). All haplogroups showed high degrees of geographic overlap with no clear physiographic separation between or within groups. Only haplogroups 1 and 6 showed a star-like structure, however, this can be attributed to the fact that these groups contain most sampled individuals, and that the rest of the haplogroups could have been underrepresented.

**Figure 3:**
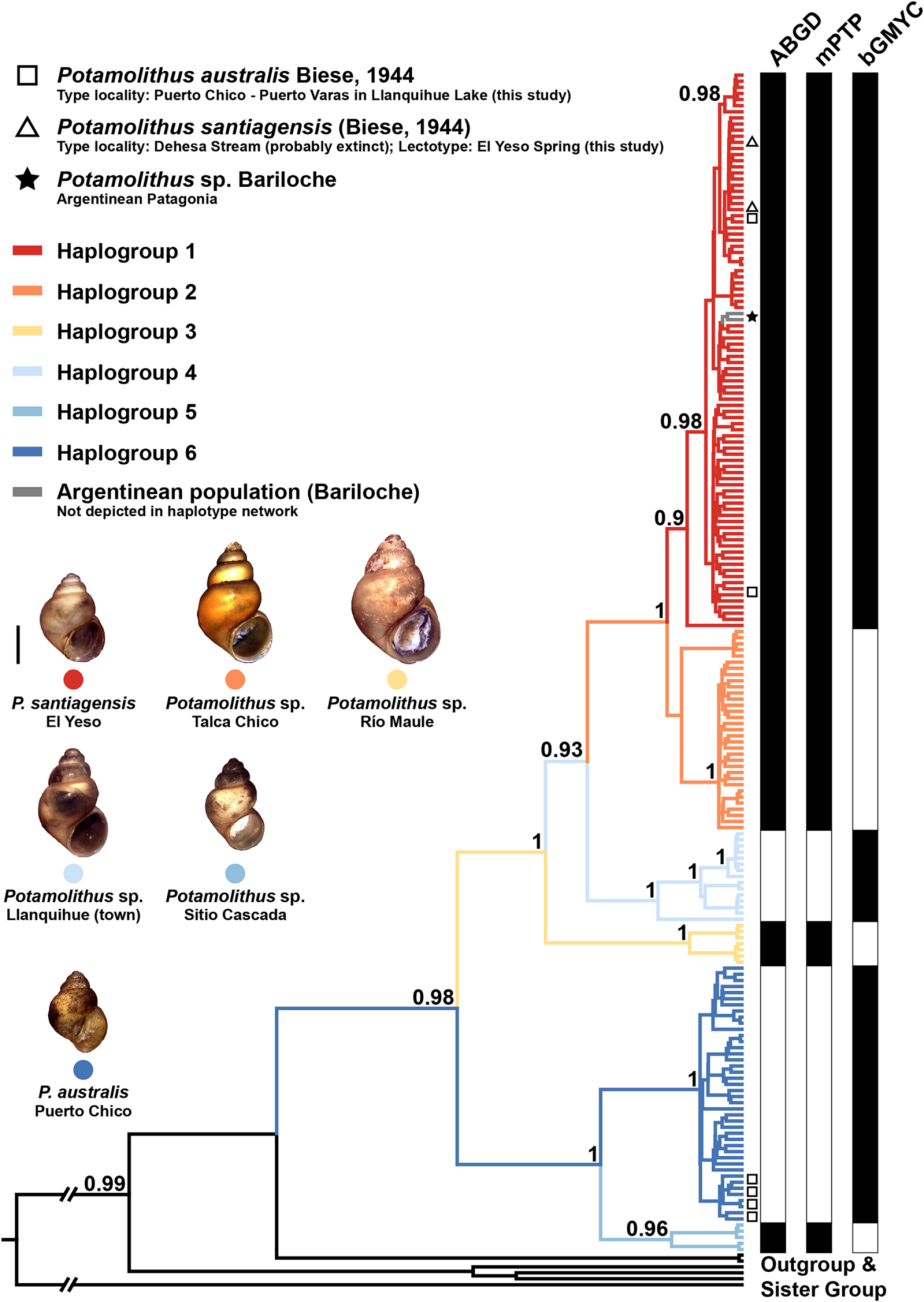
Species delimitation of Chilean *Potamolithus* populations using the COI gene. Delimitation of molecular clusters was performed using ABGD, mPTP and bGMYC. Posterior probability (> 0.9) values obtained in the Bayesian COI tree are shown at the nodes (BEAST reconstruction). Scale bar next to the shells represents 1 mm.

Both ML and BI methods recovered congruent tree topologies inferring six clades with high values of node support (Fig. S1), which correspond to the six haplogroups described by the haplotype network. These clades conform two major clades, one including haplogroups 1 to 4 and the other haplogroups 5 and 6. The species *P. elenae* (Estero Valcheta, Argentinean Patagonia) was recovered as the sister lineage of all Chilean *Potamolithus* populations. *Potamolithus lapidum supersulcatus* (Uruguay River, Argentina), *P. agapetus* and *P. buschii* (Buenos Aires, Argentina) formed a more distant lineage from the Chilean species. In addition to the Chilean populations and GenBank sequences, the phylogenetic reconstructions included two individuals from Bariloche, Argentinean Patagonia, which were recovered in Haplogroup 1 (star in Figs. 3 and S1). Genetic distances among *Potamolithus* species or haplogroups ranged from 3.1 to 11% (Table S2).

The three different species delimitation approaches were partially congruent detecting five (ABGD and mPTP) or six (bGMYC) species partitions (Fig. 3). *Potamolithus* snails from El Yeso, originally assigned to *P. santiagensis* by Biese^56^ (triangles in Figs. 1, 3 and S1), were consistently identified as a partition with the three methods of species delimitation employed here, although the ABGD and mPTP analyses included haplogroups 1 and 2 whereas the bGMYC only Haplogroup 1. Samples from Puerto Chico (Llanquihue Lake), type locality of *P. australis*, were recovered in haplogroups 1 and 6 (square in Fig. 1, 3 and S1); most samples sequenced from this locality were haplogroup 6. Following Biese^36^, individuals of Haplogroup 1 were assigned to *P. santiagensis* and Haplogroup 6 to *P. australis*. The ABGD and mPTP analyses also identified haplogroups 3 to 5 as separate partitions, while the bGMYC method haplogroups 2 to 5. Divergence times estimated in BEAST suggest that the splitting of a *Potamolithus* ancestor from *T. huonensis*, a closely related tateid, occurred about 12.4 (7.4–18.1) Ma (Fig. S2). The splitting of Chilean *Potamolithus* species occurred between 2.7 and 0.68 Ma. *Potamolithus santiagensis*, circumscribed to the distribution area covered by Haplogroup 1, split from Haplogroup 2 approximately 0.68 (0.39–0.98) Ma whereas *P. australis* from Haplogroup 5 approximately 1.5 (0.89–2.2) Ma (Fig. S2).

## Discussion

Using different molecular analyses, in the present study we showed that the biodiversity of the genus *Potamolithus* in Chile is underestimated, with more species than the two previously recognized. Our results suggest that there is cryptic diversity in the group and at least three species partitions would require validation and formal description. However, besides relying on single locus analyses, an integrative approach is recommended considering multiple loci and combined with other characters such as morphology, internal anatomy, radula, larval development and ecology, among others^59^. The phylogenetic analysis inferred at least six well-supported monophyletic groups, depicting several phylogenetic species, which is congruent with the haplogroups recovered by the network analysis.

Two of the three species delimitation methods were congruent in the number and conformation of the species partitions recovered. Five partitions were delimited using ABGD and mPTP and six with bGMYC. Previous studies have shown that the Generalized Mixed Yule Coalescent model is useful in detecting incipient speciation events^85^, when the threshold of intra- and interspecific distances is low, but on certain occasions it can overestimate the number of species due to pronounced intraspecific genetic variability^86,87^. The tree recovered in our analysis shows large branch lengths between the main clades, but short within each lineage, which is also noticeable in the haplotype network. These results suggest that the evolutionary process of the species is consistent with a recent diversification scenario (see below). Considering this, it is likely that the model implemented in the mPTP method is more appropriate to the *Potamolithus* pattern, mainly since it considers different levels of intraspecific diversity within a species as a result of their evolutionary history or uneven sampling^73^. Using a conservative approach^88^, the species delimitation analyses were consistent with the definition of five species partitions in *Potamolithus* in Chile, three of them previously undetected.

*Potamolithus australis* and *P. santiagensis* were described in the same article^36^, and partly on the same sheet, using characters from the external shell morphology and operculum, which are not entirely clear, despite they were described in different genera. Although these species have not been evaluated until now with independent morphological evidence, recent phylogenetic analyses based on COI sequences performed with samples of *P. australis* from Puerto Chico and *P. santiagensis* from El Yeso showed that they are different species^42,57,65^. Three methods of species delimitation in the present study identified these morphologically cryptic taxa as separate units, with *P. santiagensis* covering from Valparaíso Region to Tierra del Fuego in southern Patagonia, including Chiloé Island. *Potamolithus australis* has a narrower range, covering from Trupán Lake in the Bío-Bío Region to Sofía Lake in the southern section of western Patagonia in continental territory. Apart from these two species, the ABGD and mPTP methods identified three additional partitions in *Potamolithus* whereas the bGMYC method four. One of these lineages (Haplogroup 2) has a similar range to that of *P. santiagensis*, although it was not found on Chiloé Island. A second lineage (Haplogroup 4) ranges from the Maule Region to the Strait of Magellan in southern Patagonia, covering continental territory. A third lineage (Haplogroup 3) is distributed from Putagán in the Maule Region to Llanquihue Lake in Los Lagos Region, while a fourth lineage (Haplogroup 5), found inhabiting a small waterfall whose waters flow into the Puelo River in Los Lagos Region, is restricted to that single location.

The COI phylogenetic tree and network analysis of *Potamolithus* populations showed a deep genetic divergence but weak phylogenetic structure regarding the landscape physiography. Haplogroup 1 (*P. santiagensis*), Haplogroup 6 (*P. australis*), Haplogroup 2, and to a lesser degree haplogroups 3 and 4, have a wide geographic distribution in the country so the hypotheses of vicariance and speciation by basins do not explain the diversity pattern observed in the group. Moreover, we did not find evidence that phylogenetic structure corresponds to the faunistic units proposed by Stuardo & Vega^89^ for the land mollusks in continental Chile, i.e., a “northern fauna” in the north of the country and a “forest fauna” from around 38° S to Cape Horn together with a transitional zone of overlap between the units from 31 to 38° S. Furthermore, we did not find evidence of any congruence between the haplotype distribution and the biogeographic regions proposed by Dyer^90^ for Chilean fishes. Similar studies performed in different faunal groups have shown contradictory results^14^, but in all cases population differentiation was due to a mixture of evolutionary processes. For instance, in an area similar to the one studied here, a rather weak influence of geographic barriers in the differentiation of several species of the crustaceans and catfishes has been reported ^7,12,19,91^, although vicariance has also contributed to divergence in several fish species^10,12,21^.

The splitting of Chilean *Potamolithus* populations in the two major clades 2.73 (1.8–3.69) Ma is congruent with the Pliocene–Pleistocene limit about 2.59 Ma (ICS, 2008). Subsequent pulses of speciation are also congruent with the Pre-GPG in the Lower/Middle Pleistocene 2.1–1.0 Ma and the GPG in the Middle Pleistocene. The origin of *P. australis* is congruent with both dates whereas *P*. *santiagensis* with the last one; the coldest Pleistocene glaciation occurred 0.7 Ma^23^. We did not find association between contemporary levels of genetic diversity of *Potamolithus* species and the LGM. The haplotypes distributed in the north of the studied area are also found in southern Patagonia, and five of the haplogroups occur in Llanquihue Lake, a large waterbody that was completely glaciated during the early Pleistocene^23^. The presence of *P*. *santiagensis*, *P. australis* and several candidate species in southern Patagonia, an area that was covered by ice during the last ice age, may be due to the presence of refuge areas that remained in northern and/or southern Chile from which the species could have recolonized the different waterbodies once the ice melted. Colonization signals were detected in several species of sigmodontine rodents from lower latitudes in southern Patagonia^14^, with local differentiation also contributing to species diversity. Divergence times in *Potamolithus* also suggest persistence through the Pleistocene glacial cycles, similar to the pattern inferred in the sigmodontine rodents of the area^14^.

Several species of *Potamolithus* from Argentina, Uruguay and Brazil have been proclaimed with some degree of threat, mainly due to their restricted range, habitat loss, water pollution, tourism and overcollection^37,49,50,92,93^, although only seven appear classified in the IUCN Red List of Threatened Species as Data Deficient or Least Concern (*e.g.* Pastorino & Darrigran^94,95^). The conservation status of *P. santiagensis* and *P. australis* has not been evaluated until now. Although the wide range that both species occupy would mean classifying them in a lower conservation category, the presence of invasive species constitutes a serious threat, considering that in the type locality of *P. santiagensis* the species is probably extinct, possibly linked to the presence of the invasive *P. antipodarum*^44,57,65^. The occurrence of *Physa acuta* Draparnaud, 1805 in several freshwater ecosystems in Chile^96^ also constitutes a potential threat to these species, in addition to those already mentioned for other South American congeners.

## Supplementary Information

Supplementary Information.

## Data Availability

Original mitochondrial sequences obtained in the present study were submitted to GenBank (accession numbers MW916963–MW917138) (Table S1). Voucher specimens are housed at the Laboratorio de Malacología y Sistemática Molecular, Universidad del Bío-Bío, Chillán, Chile.
