# Supplementary material for: Phylogeography and molecular species delimitation reveal cryptic diversity in Potamolithus (Caenogastropoda: Tateidae) of the southwest basin of the Andes
Source: Sci Rep. 2021 Aug 3;11:15735. doi: 10.1038/s41598-021-94900-3 (PMC8333322; doi:10.1038/s41598-021-94900-3)
Supplement: Supplementary file 1 — Supplementary Information. [file 41598_2021_94900_MOESM1_ESM.pdf]

Phylogeography and molecular species delimitation reveal cryptic diversity in *Potamolithus* (Caenogastropoda: Tateidae) of the southwest basin of the Andes

Gonzalo A. Collado, Cristian Torres-Díaz & Moisés A. Valladares*

Departamento de Ciencias Básicas, Facultad de Ciencias, Universidad del Bío-Bío, Avenida Andrés Bello 720, Chillán, Chile. Grupo de Investigación en Biodiversidad y Cambio Global (GBCG), Universidad del Bío-Bío, Chillán, Chile. *Correspondence and requests for materials should be addressed to M.A.V. (email: valladares.moises@gmail.com)

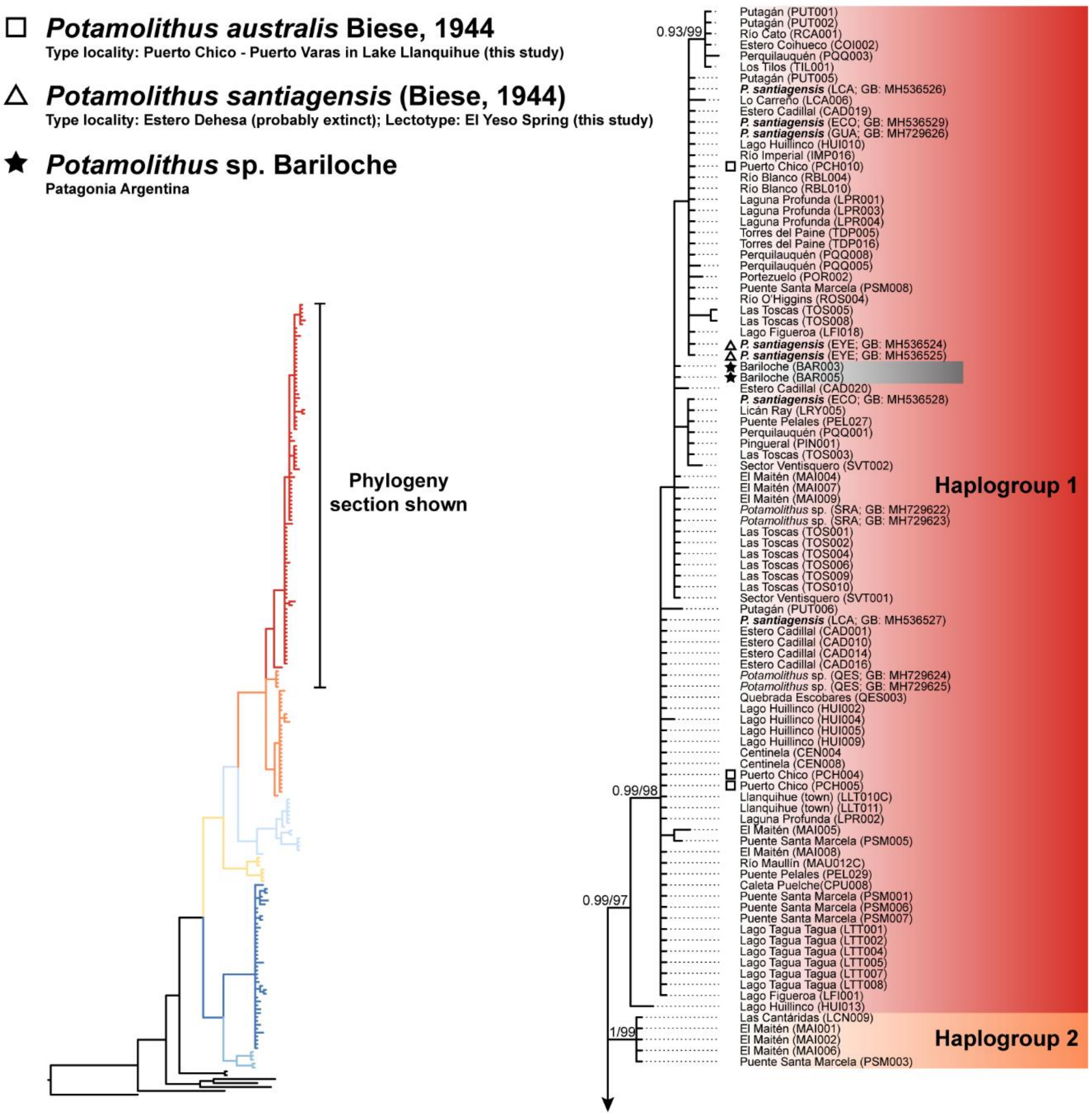

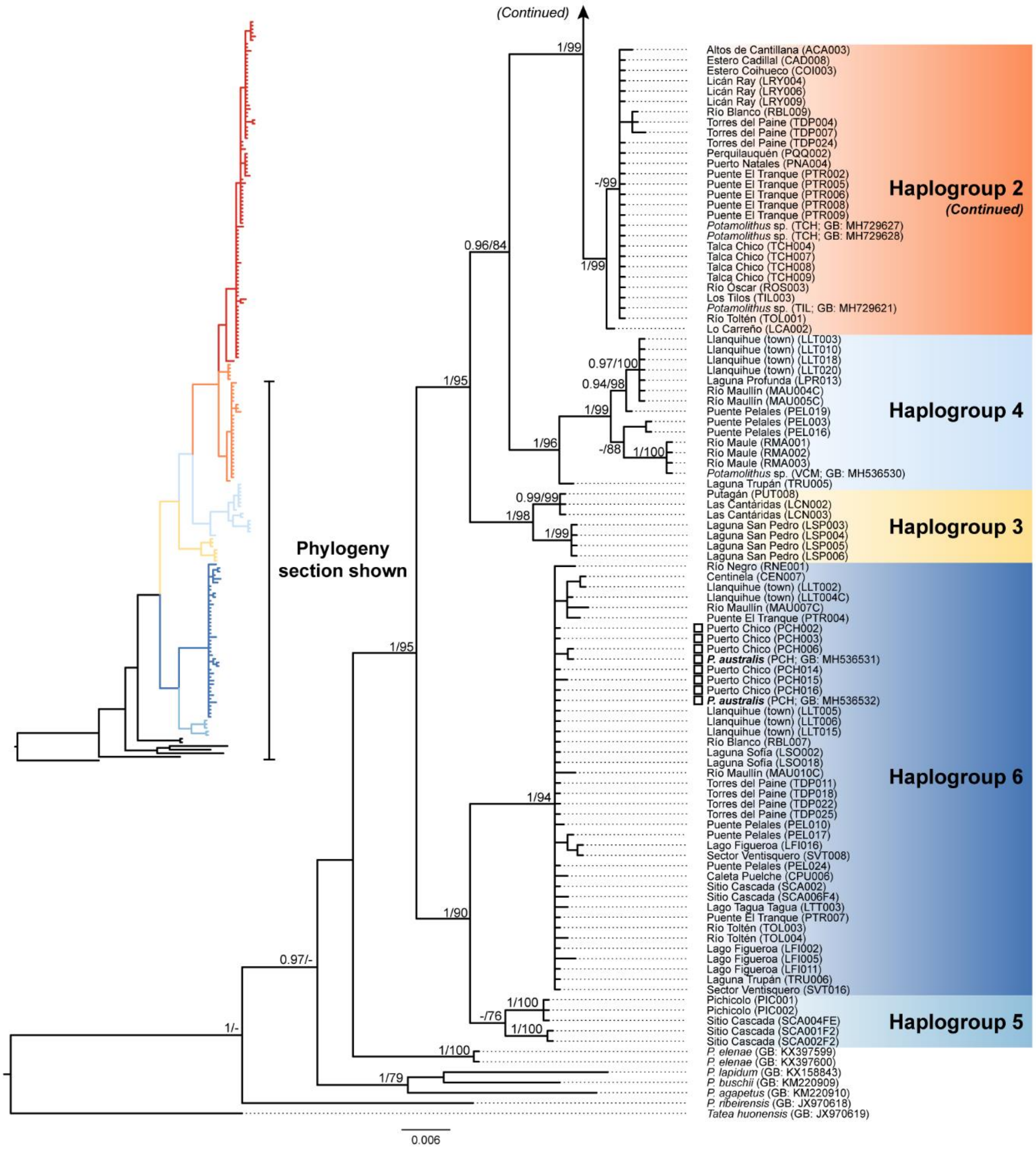

Figure S1. Phylogenetic tree based on COI gene sequences from Chilean *Potamolithus* and 7 other Tateidae representatives. Bayesian PP are given at each node followed by bootstrap values obtained in the Maximum Likelihood analysis. Haplogroups are represented by colors. Numbers after taxa names indicate GenBank isolate codes for original sequences (see Table S1) and GenBank access numbers for taxa with available information.

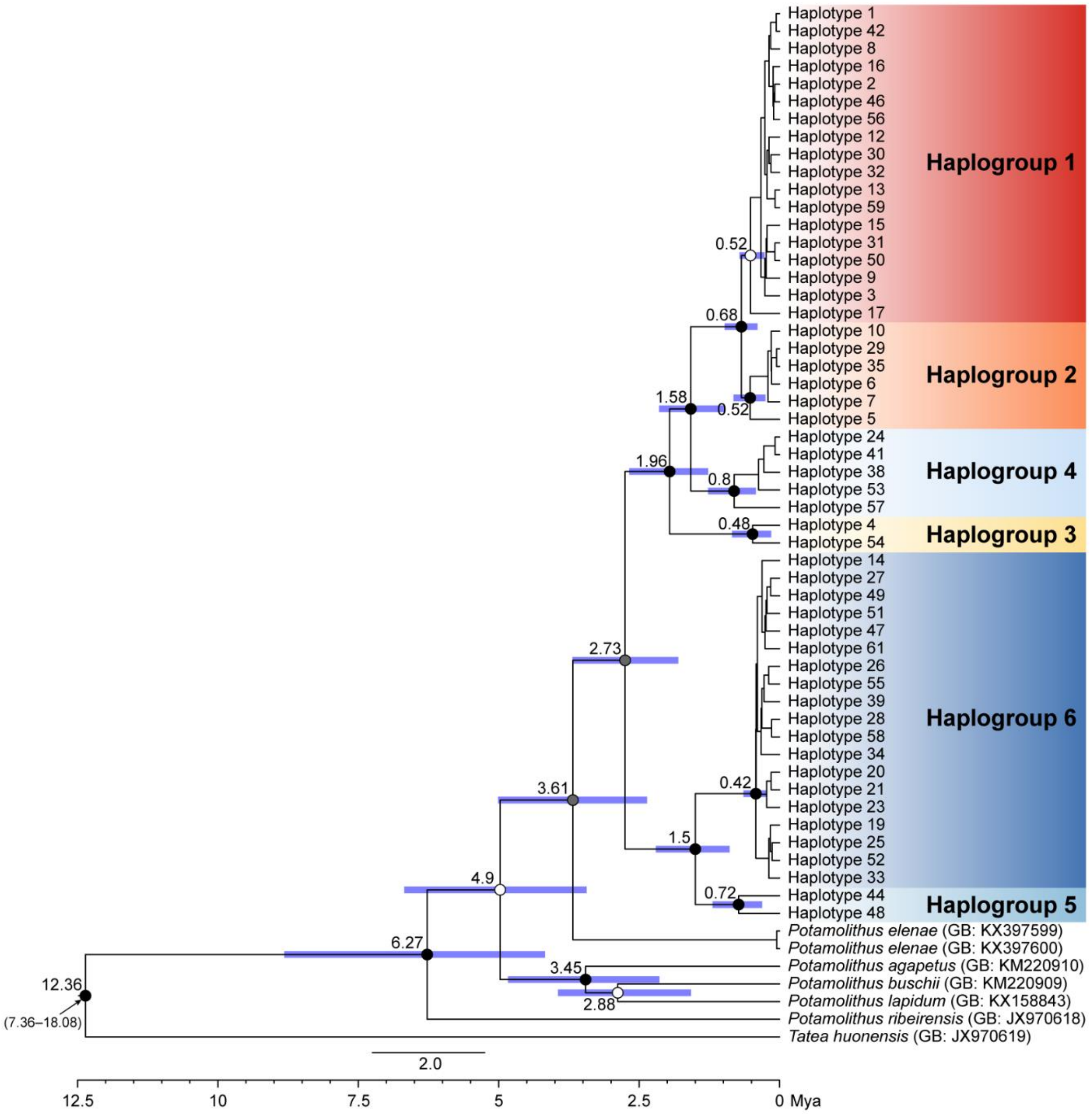

Figure S2. Divergence times estimations of Chilean *Potamolithus* based on COI sequences. Above nodes with filled circles are indicated mean divergence times estimations. Black, gray, and white filled circles indicate nodes that were recovered with posterior probability (PP) ≥0.95, 0.75 to <0.95, and <75, respectively. The 95% credible intervals for node ages are shown with transparent blue bars. Haplogroups are represented by colors. Numbers after taxa names indicate GenBank access numbers for taxa with available information (outgroup and other *Potamolithus* species).

Table S1. **Sampling localities and COI sequences of *Potamolithus* populations obtained in the present study.** Table includes locality, locality code, GenBank access number (GB), geographic coordinates (latitude and longitude in decimal degrees), number of individuals per locality (*N*) and diversity indices based on mtDNA (COI) sequences: number of polymorphic sites (*S*), number of haplotypes (*H*), haplotypic diversity (*Hd*) and nucleotide diversity (π).

Locality	Code	GB	Latitude	Longitude	*N**	*S*	*H*	*Hd*	π
Bariloche (Argentina)	BAR	MW916963-64	-41.1167	-70.7333	2	0	1	0	0
Putagán	PUT	MW916965-69	-35.8230	-71.4725	5	30	4	0.9	0.02534
Las Cantáridas	LCN	MW916970-72	-41.0947	-72.6308	3	21	1	0.667	0.0275
Altos de Cantillana	ACA	MW916973	-33.8453	-70.9982	1	-	-	-	-
Lo Carreño	LCA	MW916974-75	-34.6125	-70.9299	4	10	4	1	0.01015
Río Cato	RCA	MW916976	-36.5567	-72.0451	1	-	-	-	-
Estero Cadillal	CAD	MW916977-83	-39.9695	-73.5704	7	11	4	0.714	0.00722
Estero Coihueco	COI	MW916984-85	-36.6294	-71.8347	2	9	2	1	0.01768
Quebrada Escobares	QES	MW916986	-33.0812	-71.3037	3	0	1	0	0
Río Negro	RNE	MW916987	-41.9623	-72.4635	1	-	-	-	-
Lago Huillinco	HUI	MW916988-93	-42.6806	-73.9542	6	8	4	0.8	0.00524
Río Imperial	IMP	MW916994	-38.6997	-73.3511	1	-	-	-	-
Licán Ray	LRY	MW916995-98	-39.4848	-72.1552	4	8	2	0.5	0.00862
Centinela	CEN	MW916999-MW917001	-40.9936	-72.8792	3	36	2	0.667	0.04715
Puerto Chico	PCH	MW917002-10	-41.3270	-72.9574	11	35	5	0.782	0.03152
Llanquihue Town	LLT	MW917011-21	-41.2542	-73.0018	11	46	6	0.855	0.04179
Río Blanco	RBL	MW917022-25	-41.2303	-72.6223	4	42	3	0.833	0.04355
Laguna Profunda	LPR	MW917026-30	-53.6029	-70.9512	5	26	3	0.7	0.02181
Laguna Sofía	LSO	MW917031-32	-51.5041	-72.6893	2	0	1	0	0
El Maitén	MAI	MW917033-40	-40.9625	-72.8182	8	12	5	0.857	0.01095
Río Maullín	MAU	MW917041-45	-41.2614	-73.0025	5	49	4	0.9	0.05029

Torres del Paine	TDP	MW917046-54	-51.0468	-72.9242	9	38	4	0.778	0.04408
Puente Pelales	PEL	MW917055-62	-38.9097	-72.6229	8	44	5	0.857	0.04796
Perquilauquén	PQQ	MW917063-67	-36.2542	-71.8133	5	12	5	1	0.01063
Pichicolo	PIC	MW917068-69	-41.9947	-72.5753	2	0	1	0	0
Pingueral	PIN	MW917070	-36.5306	-72.9114	1	-	-	-	-
Puerto Natales	PNA	MW917071	-51.9680	-72.0463	1	-	-	-	-
Portezuelo	POR	MW917072	-36.7365	-72.4947	1	-	-	-	-
Caleta Puelche	CPU	MW917073-74	-41.7419	-72.6489	2	37	2	1	0.07269
Sitio Cascada	SCA	MW917075-79	-41.7286	-72.5179	5	23	4	0.9	0.02593
Puente Santa Marcela	PSM	MW917080-85	-41.6984	-72.3703	6	12	4	0.8	0.00786
Lago Tagua Tagua	LTT	MW917086-92	-41.6460	-72.1681	7	37	2	0.286	0.02077
Puente El Tranque	PTR	MW917093-99	-41.6699	-72.3171	7	38	3	0.524	0.0348
Río Maule	RMA	MW917100-02	-35.5535	-71.6992	3	0	1	0	0
Laguna San Pedro	LSP	MW917103-06	-36.8444	-73.0938	4	0	1	0	0
Talca Chico	TCH	MW917107-10	-35.4586	-71.7163	6	0	1	0	0
Río O'Higgins	ROH	MW917111	-52.7758	-69.2861	1	-	-	-	-
Río Óscar	ROS	MW917112	-52.8495	-69.7613	1	-	-	-	-
Los Tilos	TIL	MW917113-14	-32.7920	-71.0741	3	9	2	0.667	0.01179
Río Toltén	TOL	MW917115-17	-38.9745	-72.6329	3	36	3	1	0.04715
Las Toscas	TOS	MW917118-26	-36.5930	-72.0607	9	5	3	0.556	0.00349
Laguna Trupán	TRU	MW917127-28	-37.2797	-71.8189	2	26	2	1	0.05108
Sector Ventisquero	SVT	MW917129-32	-51.4313	-72.7740	4	39	4	1	0.04977
Lago Figueroa	LFI	MW917133-38	-51.3799	-72.4442	6	41	6	1	0.04126

*In this column some individuals of previous studies[57,65] were included to obtain diversity indices.

Table S2. Percent of mean sequence variation (p-distance) among *Potamolithus* species/haplogroups from COI data*.

Species/Haplogroup	1	2	3	4	5	6	7	8	9	10
1. *P. lapidum*										
2. *P. elenae*	8.8									
3. *P. ribeirensis*	10.4	9.4								
4. *P. buschii*	6.5	6.7	10.0							
5. *P. agapetus*	8.1	8.8	11.0	7.5						
6. *P. australis*	9.2	6.5	10.0	8.1	9.2					
7. *P. santiagensis*	10.6	9.0	10.8	10.2	10.2	7.3				
8. Haplogroup 2	9.4	7.9	9.8	9.0	10.0	7.3	1.8			
9. Haplogroup 3	8.8	6.5	9.4	8.4	8.3	5.9	5.3	4.5		
10. Haplogroup 4	9.4	6.9	9.4	9.0	9.2	5.9	5.1	4.7	5.1	
11. Haplogroup 5	9.8	6.5	10.8	8.4	9.8	3.1	6.7	6.3	5.5	5.5

* One sequence per species or haplogroup was used in the analysis.
